# Strategic Design and Fabrication of Engineered Scaffolds for Articular Cartilage Repair

**DOI:** 10.3390/jfb3040799

**Published:** 2012-11-14

**Authors:** Zohreh Izadifar, Xiongbiao Chen, William Kulyk

**Affiliations:** 1 Division of Biomedical Engineering, College of Engineering, University of Saskatchewan, 57 Campus Dr., Saskatoon SK S7N5A9, Canada; Email: zoi175@mail.usask.ca; 2 Department of Mechanical Engineering, College of Engineering, University of Saskatchewan, 57 Campus Dr., Saskatoon SK S7N5A9, Canada; 3 Department of Anatomy and Cell Biology, College of Medicine, University of Saskatchewan, 107 Wiggins Rd., Saskatoon SK S7N 5E5, Canada; Email: William.kulyk@usask.ca

**Keywords:** tissue engineering, scaffold design, scaffold fabrication, articular cartilage, joint repair, osteoarthritis, biomimetic, hybrid, zonal

## Abstract

Damage to articular cartilage can eventually lead to osteoarthritis (OA), a debilitating, degenerative joint disease that affects millions of people around the world. The limited natural healing ability of cartilage and the limitations of currently available therapies make treatment of cartilage defects a challenging clinical issue. Hopes have been raised for the repair of articular cartilage with the help of supportive structures, called scaffolds, created through tissue engineering (TE). Over the past two decades, different designs and fabrication techniques have been investigated for developing TE scaffolds suitable for the construction of transplantable artificial cartilage tissue substitutes. Advances in fabrication technologies now enable the strategic design of scaffolds with complex, biomimetic structures and properties. In particular, scaffolds with hybrid and/or biomimetic zonal designs have recently been developed for cartilage tissue engineering applications. This paper reviews critical aspects of the design of engineered scaffolds for articular cartilage repair as well as the available advanced fabrication techniques. In addition, recent studies on the design of hybrid and zonal scaffolds for use in cartilage tissue repair are highlighted.

## 1. Introduction

Articular cartilage is a specialized tissue that covers the ends of the bones in articulating joints. It provides a low friction, highly elastic surface [1] that can withstand dynamic compressive loads several times body weight [2]. This behavior is attributed to the complex biochemistry and physical structure of the cartilage extracellular matrix (ECM), which is secreted by the chondrocyte cells that reside therein. Unfortunately, the natural healing ability of human articular cartilage is extremely limited, which poses significant clinical challenges for the treatment of joint cartilage defects [3]. These include articular cartilage degeneration resulting from osteoarthritis (OA), a debilitating joint disease that afflicts ~70% of the population aged 65 years and older [4]. In addition, articular cartilage lesions resulting from traumatic joint injuries in children and young adults are a serious health problem with, at present, no entirely satisfactory clinical management solution [5]. Although some clinical treatments are available for articular cartilage repair [6], their success to date has been limited as they do not result in long-term correction of cartilage pathologies [1]. 

Tissue engineering (TE) aims to replace damaged articular cartilage with a long-lasting biomanufactured replacement tissue, and holds great promise as an effective treatment for joint repair. Most cartilage tissue engineering strategies incorporate three main components: a suitable biocompatible scaffold, live chondrocytes or multipotent mesenchymal cells capable of developing into chondrocytes, and a combination of appropriate bioactive molecules (e.g., growth factor proteins (GFs)) [7,8]. It is hoped the dynamic interaction of these components will generate a transplantable artificial tissue construct that integrates well with normal articular cartilage and approximates its unique biomechanical properties [9,10]. The TE scaffold is typically a three-dimensional (3-D) structure manufactured from synthetic polymers and/or natural biopolymers to provide temporary mechanical physical and biological support for the embedded chondrocytic cells; this in turn promotes their growth *ex vivo*, maintains their phenotype, and encourages their production of cartilage-specific extracellular matrix components. In addition, the fabricated scaffold exerts control over the shape and volume of the engineered cartilage tissue construct [11,12,13,14]. Accordingly, scaffold design and manufacturing techniques are critical elements for successful cartilage tissue engineering. 

Because cartilage tissue engineering is aimed at creating artificial constructs for use in joint cartilage repair, this review will begin with a synopsis of relevant features of normal articular cartilage structure and the pathology of osteoarthritic cartilage breakdown. The main body of our review will focus on critical considerations in the design of scaffolds for cartilage TE and the fabrication techniques currently available. Finally, we discuss recent and promising advances in biomimetic scaffold construction, including the use of hybrid solid polymer/hydrogel scaffolds and zonal scaffolds to manufacture cartilage tissue substitutes that more closely replicate the biomechanical characteristics and stratified organization of natural articular cartilage. 

### 1.1. Structure and Organization of Natural Articular Cartilage

**Figure 1:**
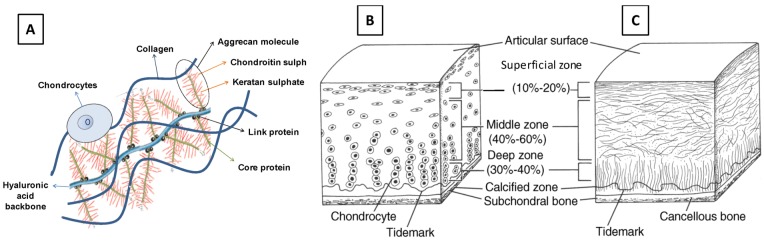
Principal components and zonal organization of articular cartilage tissue: (**A**) fibrils of type II collagen, proteoglycan complexes composed of aggrecan and hyaluronan, and chondrocytes cells; (**B**) zonal chondrocytes; and (**C**) zonal collagen fibers. ((**B**) and (**C**) are reproduced from Buckwalter *et al*. [17]).

Articular cartilage is a smooth, partially translucent tissue that covers the distal ends of bones in diarthrodial joints of the body, such as the knee and elbow. It provides a deformable, low friction surface that facilitates the movement of articulating bones within the joint and is capable of supporting high dynamic compressive loads. The unique biomechanical properties of articular cartilage are attributable to the composition of its specialized extracellular matrix (ECM), which is the secretory product of its single resident cell type: chondrocytes. Although cartilage ECM contains a plethora of molecular components [5,15], it is primarily comprised of fibrils of *type II collagen* protein together with a cartilage-specific proteoglycan, *aggrecan* (Figure 1A). The collagen II fibrils, which account for up to 60% of articular cartilage dry weight [16], provide the tissue with high tensile strength and the ability to withstand shear stresses. In contrast, the aggrecan proteoglycans (which comprise ~35% of cartilage dry weight) confer cartilage tissue with the ability to support high compressive loads. Each individual aggrecan molecule consists of a polypeptide core protein from which extend numerous covalently linked glycosaminoglycan (GAG) side chains, specifically chondroitin sulfate and keratan sulfate polysaccharides. Molecules of *link protein*, which associate with the base of each aggrecan core protein, mediate the attachment of numerous aggrecan monomers to a common long *hyaluronan* polysaccharide chain (Figure 1A). This creates huge supramolecular cartilage proteoglycan complexes embedded within the collagen II fibril network. The high negative charge densities of the chondroitin sulfate and keratan sulfate GAG side chains of the entrapped aggrecan complex create an osmotic potential that draws water into the cartilage ECM from the synovial fluid of the joint cavity and other adjacent tissues. Therefore, articular cartilage consists of up to 80% water with respect to total wet weight. Indeed, the fluid phase of articular cartilage is a critical factor for its load-bearing function [16].

Natural articular cartilage has a layered or “zonal” organization (Figure 1 B, C). Its four layers, going from the articular surface down to underlying subchondral bone, are usually termed the *superficial zone*, the *middle (or transitional) zone*, the *deep zone*, and the *calcified zone* [18]. These zones differ with respect to the molecular composition and organization of the cartilage ECM, the shape and density of the resident chondrocytes, and their mechanical properties. The superficial zone, which faces the synovial joint cavity, represents 10%–20% of total articular cartilage thickness. It is characterized by densely packed, tangentially oriented, fine collagen fibrils [19] and a relatively low proteoglycan content [20]. The superficial zone is the layer primarily responsible for bearing tensile and shear stresses [21]. The middle zone (40%–60% of articular cartilage thickness) is characterized by randomly oriented collagen fibrils [18] and the highest proteoglycan content [2], which may contribute to the higher compression modulus in this zone [22] caused by the larger osmotic water swelling effect. The deep zone (30%–40% of articular cartilage thickness) exhibits radially oriented, larger diameter collagen fibers and a lower proteoglycan content than the middle zone [20,23]. The cell density of chondrocytes decreases from the superficial zone to the deep zone, and their morphology changes from a flattened discoidal shape in the superficial zone, to a more spherical shape in the middle zone, to a slightly elongated form in the deep zone [24] (Figure 1B). The calcified zone provides a transition between the hyaline cartilage tissue of the overlying zones and the basal *subchondral bone* [5]. Within the calcified zone, the cartilage ECM is mineralized and type II collagen is replaced by a distinct type X collagen.

### 1.2. Cartilage Injuries, Osteoarthritis (OA), and Traditional Cartilage Repair Strategies

Traumatic joint injuries, abnormal joint loading, and degenerative joint diseases can all cause defects in articular cartilage tissue. Unfortunately, cartilage tissue has an extremely limited ability for self repair. This is attributed to its lack of both a vascular supply and any intrinsic mesenchymal stem cell population to facilitate tissue regeneration. Physical trauma to the knee or other articular joints can lead to several types of focal cartilage lesions, which are classified as chondral lesions, osteochondral lesions, and microfractures. Chondral defects solely affect the articular cartilage layer and do not extend to the underlying subchondral bone. In osteochondral lesions, the damage extends through the articular cartilage into the subchondral bone [5]. Microfractures, or fractures in the cartilage that are not be visible to the naked eye but affect the collagen network [25], can lead to further matrix destruction upon repeated loading [17]. Because of the inability of articular cartilage to repair itself, the initial focal cartilage damage leads to abnormal compressive loading and increased mechanical stress in the surrounding healthy cartilage, which gradually expands the area of articular damage. Over a period of years, this leads to a gradual erosion of the articular cartilage layer of the joint, resulting in osteoarthritic disease. In the end stages of osteoarthritic disease progression, the articular cartilage is totally destroyed thus exposing the subchondral bone [5]. This results in debilitating joint pain and severely reduced joint mobility. Due to the potential of traumatic joint injuries to initiate osteoarthritic disease progression, and the serious clinical consequences of late stage OA, there is tremendous interest in developing improved therapies for articular cartilage repair.

Nonsurgical treatment of OA includes activity modification, physical therapy, dietary supplements, weight loss, anti-inflammatory drugs (e.g., aspirin, ibuprofen, Celebrex), and injections of viscous hyaluronan preparations into the synovial cavity [5]. These mainly alleviate the pain and discomfort in the arthritic joint without correcting the underlying pathology. Current surgical therapies include arthroscopic microfracture, autologous chondrocyte implantation (ACI) [26], osteotomy, and arthroplasty. In microfracture surgery, damaged cartilage is removed at the site of lesion and the subchondral bone is microfractured to stimulate a healing response from subchondral bone mesenchymal stem cells and growth factors. However, the repair tissue induced by microfracture treatment is predominantly fibrocartilage that contains more type I collagen than type II collagen and has inferior biomechanical properties to articular cartilage [27]. In ACI, articular chondrocytes isolated from a healthy non-load-bearing region of a patient’s joint are expanded in tissue culture and subsequently reimplanted into the site of the cartilage lesion [28]. The tissue formed following ACI can be hyaline articular cartilage (~15% of cases) or fibrocartilage [29,30]. The scarcity of source material, donor site morbidity, and the requirement for multiple invasive surgeries limit the use of ACI [5]. Osteotomy decreases pressure in the defected area by reshaping the bone to shift the mechanical axis of bearing load to the healthier part of the joint. Osteotomy can temporarily restore knee function and decrease osteoarthritic pain, but over the long term often results in joint deterioration and eventual arthroplasty [31]. Arthroplasty, or joint replacement surgery, is the treatment for end-stage OA and involves replacement of the arthritic joint by an artificial prosthesis. Eventual loosening and deterioration of the prosthetic implant, and possible stress shielding effect induced damage to the adjacent bone, are some limitations of arthroplasty surgery. Moreover, arthroplasty is unsuitable for child and adolescent patients whose skeletons are still growing and who require a long-term solution [5]. Indeed, the common problem in most current therapies is their inability to provide long-term relief and resumption of activity [5]. The aim of cartilage tissue engineering is to promote long-lasting, functional repair of defective articular cartilage lesions through the development and *ex vivo* manufacture of implantable artificial cartilage tissue substitutes.

## 2. Scaffold Design for Cartilage Tissue Engineering

Design of TE scaffolds for cartilage repair generally includes customization of biochemical and physical properties for better engineering of cartilage tissue constructs [32]. Biochemical design concerns chemical composition and biological properties of the scaffold, which mainly affect the cellular behavior and activity. Physical design concerns the internal and external scaffold architecture, mechanical properties, and degradation properties. This review discusses physical design considerations of engineered scaffolds with a brief discussion of the typical backbone materials used for cartilage tissue engineering. 

### 2.1. Scaffold Backbone Materials

**Figure 2:**
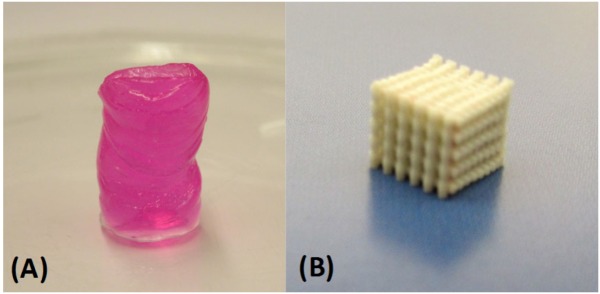
Examples of (**A**) hydrogel; and (**B**) solid scaffolds.

The scaffold material is one of the main design factors to be considered in scaffold-based cartilage TE. The chosen material should meet several criteria, including biocompatibility, mechanical strength, cell affinity and ability to promote cartilage tissue formation, and adjustable biodegradability. The mechanical properties of the material are particularly important for cartilage TE applications due to the load-bearing nature of the target tissue. More precisely, a scaffold material must maintain its structural integrity and stability during fabrication, clinical handling, and fixation at the implant site [33]. It should also protect the embedded cells from harmful mechanical stresses and withstand the *in vivo* loading environment until the newly formed tissue can assume the load-bearing function. Furthermore, it should provide a desirable environment for biological activities such as cell attachment, proliferation, differentiation, and cell–cell interaction [5,32,33]. Hydrogels (highly hydrated polymer networks) and solid polymers are typical scaffold materials and have been widely investigated for cartilage tissue engineering (Figure 2). 

Hydrogels are easily prepared and embedded with chondrocytes, which retain their phenotype and morphology through impregnation [34,35]. Hydrogels can be made of a wide variety of biomaterials, including natural materials, which may be carbohydrate-based (e.g., alginate, agarose, chitosan, hyaluronic acid (HA)), protein-based (e.g., fibrin glue, collagen type I and II, silk) [5,28], or some combination of the two, and synthetic materials, such as poly(hydroxyethyl methacrylate), polyethylene glycol and its derivatives, or poly(vinyl alcohol) (PVA) [36]. Hydrogels exhibit characteristics similar to soft tissues and therefore provide a supportive matrix for chondrocyte activity and cartilage ECM secretion both *in vitro* and *in vivo* [37,38,39,40]. High efficiency of cell encapsulation and uniform cell distribution within the hydrogel are advantages that influence the quality of formed tissue. However, hydrogels have very weak mechanical properties that limit their application for cartilage TE [41]; for example, their compression moduli can range from 10% to 20% [42,43] to 50% [44] of natural cartilage values. Limited control over shape and internal structure is another issue that makes architectural design of hydrogel scaffolds difficult. A comprehensive review of hydrogel scaffolds for cartilage TE is given in [45]. 

Polyester-based solid scaffolds are generally created from biocompatible synthetic materials and have superior biomechanical properties to those based on hydrogels [1,5]. Furthermore, these materials allow easier fabrication of scaffolds with designed shapes and internal architectures. Some frequently used synthetic polymers in cartilage TE include poly (glycolic acid) (PGA), poly(lactic acid) (PLA), poly(lactic-co-glycolic acid) (PLGA), poly-caprolactone (PCL), and poly(ethyl glycol) (PEG), all of which received FDA approval more than 20 years ago. Created by chemical processes, synthetic polymers allow for easier customization of material properties, e.g., mechanical and degradation properties, compared to hydrogel materials. PCL- and PGA-based scaffolds with exactly the same structure exhibit markedly different mechanical properties; for example, the aggregate modulus of a PCL-based scaffold was 0.787 MPa compared to 0.173 MPa for a PGA-based scaffold [46]. Mechanical modulus values of scaffolds made of poly(glycerol sebacate) (PGS) polymer, a newly developed biomaterial, can be increased to the range of native articular cartilage by material modifications that include changing the molar ratios of glycerol:sebacic acid and increasing the polymer curing time [47]. Although their adjustment can improve scaffold mechanical strength, material properties should still allow for gradual hydrolytic attack and degradation of the temporary scaffold [48] and replacement by neocartilage. Although mechanically suitable for cartilage TE, solid synthetic scaffolds have shown less affinity for cell adhesion and activity than hydrogels [28]. Different techniques have been used to improve surface cell adhesion and bioactivity of synthetic polymers, such as blending or copolymerization with hydrophilic/hydrophobic materials including chitosan [49], polymethacrylic acid [50], fibronectin and collagen [51,52], and chondroitin sulfate molecules [36]. A review of polymeric materials for cartilage tissue engineering is given in Puppi *et al*. [53]. Recently, decellularized tissue materials (*i.e*., shattered natural cartilage ECM) have been used to create solid scaffolds for cartilage TE [54,55,56]; natural ECM proteins and structures present in the decellularized scaffolds promote good cell affinity and ECM formation. 

### 2.2. Scaffold Physical Architecture

**Figure 3:**
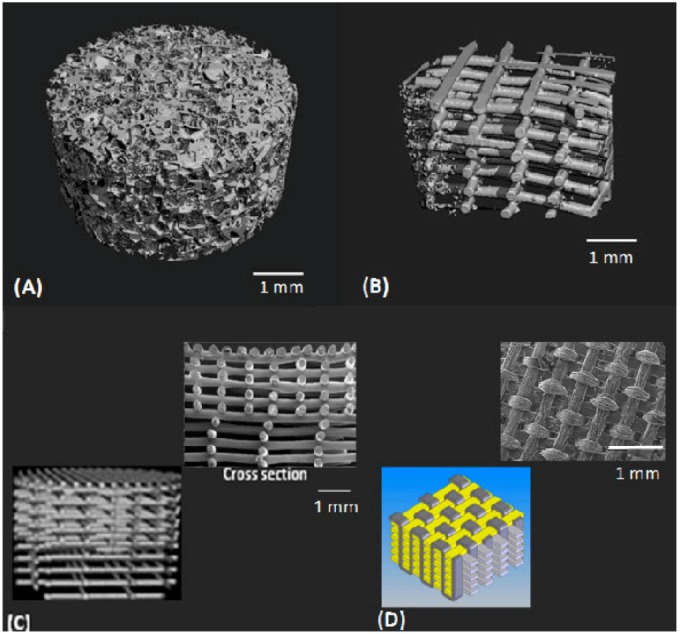
Different scaffold structural designs for cartilage tissue engineering: (**A**) 3D sponge [57]; (**B**) fibrous [57]; (**C**) gradient [61]; and (**D**) woven [46].

As natural cartilage tissue originally develops in a 3D environment, there are distinct advantages of using 3D *vs.* 2D scaffold structures, including better maintenance of chondrocyte morphology and differentiation [48,57] and higher expression of genes that regulate cell activities and ECM production [58,59]. The most commonly used 3D scaffold architectures in cartilage TE are porous 3D sponges and nonwoven fibrous structures (Figure 3A,B) [60]. Gradient fibrous structures [61], whose architectural properties vary through the depth of scaffold (Figure 3C), and woven architectures [46] (Figure 3D) have also been developed for cartilage TE. Different design parameters within these architectures, including pore size and geometry, pore distribution, pore accessibility and tortuosity, and porosity, play significant roles in the morphology, composition, mechanical properties, and functionality of the neocartilage [48,62,63,64]. 

The influence of scaffold pore size on cartilage formation has been studied [65,66,67]. The presence of both macro and micropores are important in 3D scaffolds, as macropores (>50 µm) promote cell migration [68] and micropores promote cell–cell interaction and mass transport, which improve tissue formation especially *in vivo* [69]. Titanium alloy constructs with a small average pore size (13 μm) form thicker cartilage tissue with significantly greater proteoglycan and cell density than those with larger pore sizes (43 and 68 μm) [67]. Larger pore sizes (400 μm) in nonwoven fibrous scaffolds cultured with cells result in a significantly larger GAG content compared to constructs with 100 and 200 μm pore sizes [70]. Lien *et al*. [71] show that pore sizes between 250 and 500 μm in scaffolds are appropriate for chondrocyte proliferation and ECM secretion. Cell response and chondrogenesis may vary with pore size and influence different components of tissue formation, such as cell phenotype and activity as well as the amount and composition of ECM; small pore sizes might help maintain chondrocyte phenotypes, as chondrocytes are more likely to differentiate when the pore size is about 30 times the cell diameter (10–15 µm) [72,73,74]. On the other hand, larger pore sizes increase the extension of ECM. In other words, small pore sizes increase the tendency for cell proliferation, while large pore sizes benefit the formation of ECM [71]. Gradient structures [61] or composite scaffolds (micro to macropore structures [69]) that exhibit a range of pore sizes can better facilitate both chondrocyte activity and secretion of ECM. In addition to pore size, scaffold fiber size affects the cell activity. Relatively large fiber diameters (hundreds of micrometers) in fibrous scaffolds negatively influence cell function because they present as a more two dimensional (2D) attachment surface for individual cells [21]. Chondrocytes seeded on nanofiber meshes show better chondrogenesis than on larger fiber scaffolds [75].

High degrees of porosity and pore interconnectivity are essential factors in the design of scaffolds, because they allow for initial cell or cell–carrier substance impregnation into and through the scaffold, further cell–cell interactions, mass transfer of nutrients and metabolites, and tissue growth [32]. Highly porous substrates provide for more cell attachment (about four times) than less porous substrates and result in more cartilage tissue formation [76]. Although this might be due to a greater chance of nutrient and metabolite transfer in more porous scaffolds, the exact factor(s) responsible for higher cell spreading and ECM production in the highly porous substrate are not known. Practically speaking, there is a compromise between porosity and mechanical properties of the scaffold, where the degree of stiffness tends to decrease as porosity increases [77]. As a result, scaffolds should be designed to provide an interconnected pore network with enough overall porosity while maintaining sufficient strength and stiffness [69,78]. Sponge scaffold designs generally have higher porosities than fibrous scaffolds [57], although the porosity of fibrous scaffolds can be precisely controlled in the fabrication process [61]. Woven scaffolds have compact structures with low porosity (70% [79]) and limited interconnectivity [21], which may cause inhomogeneous cell seeding and tissue growth as well as reduced nutrition transfer throughout the scaffold [46,80,81]. Gradient structures with varying porosities might allow tailoring of both mechanical and architectural properties with minimal compromise. 

Pore interconnectivity can influence the ability of a scaffold to support chondrogenesis as well as the quality of formed tissue, even in scaffolds with similar porosities [57,82]. Interconnected structures with open pores are more capable of facilitating homogeneous cell seeding and better nutrient dispersion throughout the construct [21]. Lack of sufficient pore interconnectivity can cause inhomogeneous chondrogenesis, with cartilage formation only evident on the peripheral boundaries of TE constructs [83,84]. Sponge scaffold architecture is largely controlled by the fabrication process rather than design parameters [85], and thus there is no control over the interconnectivity of the pores. A comparison of 3D sponge and organized fibrous scaffolds shows that the sponge scaffold contains random pores with tortuous paths, a lower average pore size (about five times smaller), and a higher specific area [57]. In addition, sponge architectures have a higher chance of pore blockage (filtration effect) than fibrous architectures. As the diffusion coefficient of a structure is directly proportional to porosity and inversely proportional to tortuosity, diffusion of nutrients is less in sponge structures than organized fibrous structures despite both architectures having similar porosities. This difference may not result in different cartilaginous tissue growth *in vitro* [57]; however, *in vivo* implantation revealed more chondrogenesis in the organized fibrous scaffold that had a denser and more homogeneous structure. These results demonstrate the effect of scaffold architecture as well as the importance of considering differences between *in vitro* and *in vivo* environments when designing scaffolds.

The design of pore distribution using natural cartilage as a model has also been investigated in oriented porous polymer scaffolds [86], fiber-reinforced scaffolds [87], and gradient pore size structures [61]. In the latter study, growth of cartilaginous tissue in fibrous scaffolds with three different fiber spacings along the length of scaffold (large to small spacing from bottom to top) was investigated for mimicking the zonal structure of natural cartilage. Results indicate that an anisotropic scaffold architecture can promote inhomogeneous tissue formation; however, it cannot produce zonal cartilage matrix similar to natural tissue. Different parameters in the architectural design of a scaffold may not have similar effects on all components of engineered cartilage, and different combinations of these parameters may differently influence cartilage tissue formation. For instance, scaffold architecture and pore size may not influence the formation of collage type II [57,66] and total collagen content [67]. However, chondrocyte proliferation and GAG content are improved in scaffolds with small pore sizes (<20 µm) (more cell–cell interaction) [65]. Thus, it is important to investigate how the various components of scaffold architectural design influence the individual biological properties of the engineered cartilage construct. Table 1 summarizes some of the architectural properties of scaffolds, with ranges of associated values for two commonly studied designs (sponge and fibrous) in cartilage TE applications. 

In contrast to solid scaffolds, hydrogels do not provide much flexibility for creating structures with defined internal architectures. Few efforts have been able to develop architecturally designed hydrogel scaffolds [88,89,90,91] and, therefore, the effect of architectural parameters on chondrogenesis, as has been considered for solid scaffolds, is yet to be determined. However, the significant influence of other hydrogel scaffold design parameters, such as macromere molecular weight and wt % concentration [92,93], as well as the structure of the polymer network (e.g., mesh size) [94,95,96], on the synthesis and distribution of engineered cartilage ECM has been investigated. Higher wt % concentration of HA hydrogels can better upregulate collagen type II and proteoglycan content [92]. However, the higher density of the hydrogel network impedes the distribution of deposited ECM, which interferes with the mechanical properties of the formed tissue construct [93]. The structure of the hydrogel network, controlled by the polymer chemistry and gelation mechanism [94,96,97] can dictate the spatial distribution of formed ECM by influencing the diffusion of nutrients and the degradation profile. To engineer a functional cartilage tissue construct, a fine balance among these design parameters must be achieved. A review of design aspects for hydrogel scaffolds along with examples of successful hydrogel systems for impregnation of chondrocytes are given in Nicodemus and Bryant [97].

**Table 1:** Typical architectural properties of sponge and fibrous scaffold structures.

Scaffold	Avg. Pore size (µm)	Porosity	Surface area (mm^2^/mm^3^)	Interconnectivity	Fiber size	References
**Sponge**	50–500	48%–95%	55.6	<100%	--	[37,98,99,100,101,102]
				(low cut off value*)		
**Fibrous**	100–1650	48%–87%	16.5	100% (high cut off value)	30–250 µm	[21,34,61,70,85,103,104,105,106]
Macro fibers						
	20–80	84%–90%	--	--	60–100 µm	
Micro/nano fibers						

*cut off value; fraction of total pores that are 100% interconnected.

### 2.3. Mechanical Properties

The load-bearing nature of the joint environment in which the engineered scaffold/construct will be implanted must be considered when designing the mechanical properties of the scaffold. Design considerations with respect to mechanical strength will depend on whether the scaffold will be used *in vitro* or *in vivo*. If the scaffold will be implanted into an articular joint *in vivo* shortly after fabrication, its mechanical characteristics should ideally match those of natural cartilage to support the loads encountered in the joint. The peak force applied to knee cartilage during normal physiological loading ranges from 1.9 to 7.2 times body weight [107], which corresponds to about 0.84 to 3 MPa for a 70 kg person [108]. If the scaffold is designed to initially promote engineered tissue growth *in vitro* before implantation *in vivo*, it may not require the same level of mechanical strength as natural cartilage. This is because the scaffold will largely act as a supportive environment to help formation of the tissue construct. Rather, the newly formed tissue or neocartilage–scaffold construct (partially developed cartilage) must achieve the required mechanical properties to replace the damaged tissue *in vivo* [5]. 

In addition to the initial strength of the backbone material, architectural properties can play an important role with respect to the overall mechanical properties of the scaffold. The dynamic stiffness of 3D fibrous scaffolds [57] is higher than 3D sponge scaffolds, and close to that of bovine articular cartilage [85] and human knee cartilage [109], even when both scaffolds are made of exactly the same material. Poly-l-lactide acid (PLLA) sponge scaffolds (300 µm pore sizes and 90% and 95% porosities) and fibrous scaffolds (1 mm pore sizes and 85% porosity) can all exhibit mechanical properties (compression Young’s modulus) similar to that of natural cartilage [110].

Mechanical properties of scaffolds are also influenced by the variation of architectural parameters, such as porosity [69,111], pore size [70,111], pore shape [112,113], fiber diameter [1], and fiber spacing [85]. Fibrous scaffolds with different macropore and macro/micropore porosities have varying mechanical properties [69]. El-Ayoubi *et al*. [69] demonstrated the design of scaffold architectures that result in similar mechanical properties to bovine articular cartilage and a good environment for cell attachment and activity. Sponge scaffolds with macropores ranging from 300 to 500 µm have varying mechanical properties, with those of lower porosity best resembling the performance of rabbit articular cartilage in compression and stress relaxation tests [111]. 

The effect of pore size on the mechanical properties of woven chitosan-HA copolymeric fibrous scaffolds was studied by Yamane *et al*. [70]. Chondrocyte-seeded scaffolds with the smallest pore size (100 µm) had a larger compression modulus than scaffolds with 200 or 400 µm pore sizes. However, after 28 days of cultivation, the scaffolds with the smaller pore sizes had reduced compression moduli while the two others (specifically 400 µm) had increased compression moduli and significantly enhanced ECM production. Thus, the scaffold with the larger pore size presents a better environment for developing a stronger tissue substitute. Increased ECM content can also be accompanied by improved mechanical properties [114,115]. The influence of pore shape on the mechanical properties of cartilage TE scaffolds [112,113] and constructs [113] as well as chondrogenesis [116,117] has also been studied. Chondrocytes produce more robust ECM (higher sulfated GAGs) in the presence of ellipsoidal pores than cubical pores. Jeong and Hollister [113] show that a 3D spherical pore-shaped scaffold enhances chondrogenic activity compared to cubical pore shapes. When the porosity and surface area of the scaffolds were kept similar, increased chondrogenesis in spherical pore-shaped scaffolds was attributed to the lower permeability, subsequent higher cell aggregation and GAG retention, and lower oxygen tension within the scaffold. The significant influence of a pore shape on scaffold mechanical properties (i.e., stiffness and nonlinearity) and *in vivo*-generated cartilage tissue constructs has also been demonstrated [113]. Scaffolds with pore size gradients [61] or oriented structural parameters [46] exhibit associated anisotropic mechanical properties, which can be employed to mimic anisotropic biomechanical properties of natural articular cartilage. These observations demonstrate the importance of simultaneously considering different factors for achieving the most appropriate design for cartilage TE scaffolds. 

Hydrogel scaffolds have intrinsically weak mechanical properties for *in situ* placement in cartilage. Although architectural properties, similar to those used for solid scaffold design, may not be used for modifying hydrogel properties, other strategies have been investigated for improving the initial mechanical properties of hydrogels; this includes mixing with other molecules or polymers [118,119,120,121], using cross-linking agents [122], and changing macromere chemistry, molecular weight, and polymer concentration [96,121,123]. In the latter two studies, the compression modulus of the scaffolds created ranged from 0.005 to 2.6 MPa, comparable to that of healthy human articular cartilage. However, these modification methods can introduce chemical toxicity and impair nutrient diffusion [124]. Hydrogels with low initial mechanical properties can promote the formation of constructs with high mechanical strength (*i.e.*, compressive and dynamic modulus) compared to hydrogels whose mechanical strength is initially higher [93]. As a result, design considerations for the initial mechanical properties of hydrogels should be balanced with parameters that control the synthesis of tissue ECM. 

The design of TE scaffolds for cartilage repair should also consider mechanical compression, tensile, and shear properties to achieve functionality similar to natural cartilage. However, scaffolds in most studies have been designed and tested with respect to one or two mechanical properties at the expense of others. Compression properties have received more attention in scaffold mechanical design than tensile and shear properties. 3D-woven structures made of polymeric biocompatible yarns [46,70] demonstrate considerable improvements in some mechanical properties, such as nonlinear, anisotropic mechanical properties, as well as high tensile strength and stiffness. One potential research area for the mechanical design of scaffolds is the investigation of the optimum architecture for improved mechanical properties. A study of the molecular/cellular structure and organization of the human body’s musculoskeletal/cytoskeletal system shows that the specific architectural framework of a structure (e.g., a tensegrity structure) can maximize its strength, flexibility, and structural integrity with minimum employed mass [125]. Such framework architectures could be investigated with respect to TE scaffolds for cartilage applications. 

Overall, the design of scaffolds with adequate mechanical strength is challenging. Currently available cartilage TE scaffolds still require mechanical property improvements, as mostly remain inferior to natural human cartilage with respect to supporting the loads at the damaged site. Moreover, excellent mechanical properties of TE scaffolds do not guarantee the growth of cartilage tissue substitutes with mechanical properties similar to natural cartilage. To date, the mechanical properties of most engineered hyaline cartilage substitutes remain largely inferior to natural cartilage [68,126,127,128,129]. For instance, mechanically stimulated agarose disks with seeded cells had an aggregate modulus of 0.1 MPa (0.1–2.0 MPa for natural cartilage) [68], and agarose and chitosan constructs cultivated for 20 days had compression moduli of 0.028 and 0.011 MPa, respectively [130], both of which are lower than values for native cartilage (Table 2). Although scaffolds with sufficient mechanical strength can protect the healing site and may indirectly influence the quality of neocartilage, numerous factors should be considered with respect to the regeneration of native-like engineered cartilage in terms of mechanical properties. Furthermore, changes in mechanical properties during *in vitro* culture or after *in situ* implantation are very important considerations for scaffold design. Such changes are closely linked to the degradation rate and profile of the scaffold *in vitro* or *in vivo*. This relationship has been explored to advance designs that consider changes in scaffold mechanical properties in culture or during *in vivo* healing time. Time-dependent mechanical properties have been modeled during degradation based on the change in microstructure and/or material properties of the scaffold [131]. A profile of change in scaffold mechanical properties has also been designed [132] based on a proposed profile of degradation [48]. Consequently, the variation of scaffold mechanical properties while *in vitro* or *in vivo* can be controlled/customized using factors that govern the degradation process. Table 2 shows the range of some biomechanical properties for natural human cartilage and cartilage TE constructs. Different mechanical tests (e.g., compression, tensile, and shear tests) have been used to evaluate the mechanical function of TE cartilage scaffolds, constructs, and natural cartilage. These mechanical properties have been described in detail and the associated testing procedures for cartilage TE applications have been comprehensively reviewed in Little *et al*. [133].

**Table 2:** Biomechanical properties of natural human cartilage and cartilage tissue engineering constructs with associated ranges.

Mechanical properties	Healthy human articular cartilage	References	Cartilage TE construct	References
Tensile Young’s modulus (MPa)	5–25	[134,135,136]	0.089–400	[34,46,137,138]
Ultimate tensile stress (MPa)	15–35	[139,140]	5.27–85	[34,46,137]
Compression Young’s modulus (MPa)	0.24–0.85	[141,142,143]	0.005–5.9	[46,70,123]
Complex shear modulus (MPa)	0.2–2.0	[144]	0.023–0.11	[46,145]

### 2.4. Degradation Properties

Scaffold degradation is an important aspect in the design of TE scaffolds as it can affect the formation and/or functionality of new tissue, as well as the response of host tissue [146]. Ideally, the rate of scaffold degradation should be proportional to the rate of tissue formation to ensure sufficient mechanical support at the defect site until the new tissue can fully assume load-bearing function [48]. The implementation of this strategy is practically challenging. The degradation properties of scaffolds depend on and can be modified by variables including biomaterial type and composition [18], surface chemistry [147], scaffold local environment [132], and architecture [148]. These factors can be used in the design of scaffolds to customize their degradation behavior during cartilage tissue growth *in vitro* or *in vivo*. Manipulation of the scaffold material has been a common strategy to control degradation behavior. The degradation behavior of naturally-derived scaffolds is largely influenced by the intrinsic properties of the material, over which there is very limited control. Some strategies used for controlling the degradation rate in naturally derived solid/hydrogel scaffolds include change of material deacetylation degree [5,149], chemistry [150], molecular weight, wt % concentration [151,152], and combination with other polymers [94]. For polymeric scaffolds, strategies such as alteration of polymer/copolymer composition and molecular weight [153,154], crystallinity [155], and incorporation of additives [155] have been undertaken to modify degradation behavior. Local environmental parameters such as temperature and pH are also factors that affect material degradation [155,156] by accelerating/decelerating hydrolysis processes. Mechanical loading (i.e., dynamic loading) also accelerates degradation of scaffolds *in vitro* [157], which is an important factor to be considered with respect to the design of scaffolds for *in vivo* cartilage TE applications.

Architectural properties affect the degradation of polymeric scaffolds [158,159,160], with scaffolds of higher porosity or smaller pore sizes degrading more slowly than those with lower porosity or larger pore sizes. This is attributed to the greater thickness of pore walls and the associated earlier autocatalysis hydrolysis inside the struts [156], and domination of bulk degradation over surface degradation. Although the exact involvement of each degradation mechanism has not been quantitatively determined, both pore wall thickness (bulk degradation) and surface area (surface degradation) of the scaffold should be considered with respect to scaffold degradation. For hydrogels, the structure of the gel, namely the mesh size of the cross-linked network, influences the degradation profile [161], with highly cross-linked hydrogels (smaller mesh size) exhibiting longer degradation times. The difference between scaffold degradation behavior in *in vitro* and *in vivo* microenvironments [162,163,164] indicates the importance of considering the properties of the actual *in vivo* microenvironment (e.g., the presence of enzymes) when strategies for scaffold degradation control are developed for cartilage TE applications. 

## 3. Fabrication of Designed Scaffolds

Creation of TE cartilage scaffolds can be as challenging as scaffold design. The fabrication process must generate a scaffold with a reproducible architecture, which can function as designed for a specific period of time in the load-bearing environment of a joint (if implanted shortly after implantation) [32,48]. The choice of manufacturing method can influence different characteristics of the scaffold, including structural architecture, mechanical properties, biocompatibility, and biochemical properties (cell/bioactive agent incorporation) [165].

Current fabrication techniques include solvent casting, particulate leaching, melt molding, phase separation, freeze-drying, and gas foaming [48,58,166]. Some methods, such as freeze-drying, can generate porous scaffolds containing both small (e.g., 15 to 35 µm) and large (>200 µm) pore sizes [167,168]. Using these conventional techniques, scaffold properties can only be controlled by process and equipment parameters rather than design parameters [85]. Thus, scaffold architectural design parameters (e.g., pore size, geometry, interconnectivity, distribution) cannot be precisely controlled or customized. Extensive use of highly toxic solvents and extreme processing conditions (e.g., high temperature, pressure) in most of the current fabrication methods [48] are disadvantages for advanced designs and strategies, such as incorporation of viable cells and bioactive molecules during scaffold fabrication (termed biofabrication) [165]. Detailed information along with the pros and cons associated with these current techniques are summarized by Hutmacher [48] and Sachlos and Czernuszka [169].

Textile technologies, including classical nonwoven textile and electrospinning, are other methods for fabrication of highly porous scaffolds from polymer fibers. Electrospinning methods build scaffolds from micron/submicron fibers that are similar to the size of collagens in the ECM of cartilage [34]. Briefly, the electrospinning process involves creations of an electrically charged jet of polymer that is ejected across a high voltage electric field. The spinning polymer fibers lay randomly on a grounded collecting screen to create a scaffold. These fine-fiber-based electrospun structures can have better mechanical properties than other fiber-based scaffolds [34,170,171], such as similar tensile properties to human cartilage [171]. Although electrospun scaffolds can have superior stiffness and tensile strength, more studies are required to test their mechanical performance (e.g., compression, shear strength) in both dry and hydrated conditions. Some advantages and limitations of electrospinning techniques that should be considered in designed-based scaffold fabrication are summarized in Table 3. In terms of design-based scaffold fabrication, electrospinning has the capacity to create meshes with aligned nanofibers that resemble the anisotropic structure and mechanical properties of cartilage tissue [172,173,174]. Although electrospinning methods can create oriented fibers, to date the anisotropy of the electrospun fibers is only controllable in one direction; therefore, scaffolds with greater architectural complexity (e.g., gradient structure, spatially controlled properties) cannot be easily created. Focused melt electrospinning has introduced some improvements by enabling deposition of patterned nanofibers [175], which could be advanced to fabrication of 3D scaffolds with more complex, designed structures for cartilage TE. Melt electrospinning has also been successfully tested for direct deposition of nanofibrous polymers onto the cells *in vitro* [176], which has the potential to be improved for biofabrication of scaffolds with incorporated cells and/or bioactive molecules for cartilage TE applications.

Additive manufacturing (AM) techniques, also known as rapid prototyping (RP), is a computer-controlled fabrication technique that enables reproducible fabrication of scaffolds with designed internal and external architecture [32,169]. In brief, the scaffold model, designed or customized (*i.e.*, based on medical images of defect area) by computer-aided design (CAD) software, is physically built layer by layer using selective materials as specified by a computer program [32,48]. Two main categories of AM techniques used for cartilage TE include extrusion-based (e.g., melt/dissolution plotting [57,177,178]) and particle/polymer bonding (e.g., stereolithography [179], selective laser sintering [180]). The main advantages of the AM techniques in strategic TE of cartilage include developing scaffolds with a range of designed mechanical and architectural properties, including copolymer composition, porosity, and pore geometry with high precision [85,181,182,183]. These capabilities of AM approaches have made strategic study of the cellular response to scaffold architectural design possible [61,184,185,186]. Some modern AM techniques, such as 3D printing, 3D plotting, sterolithography, and laser-assisted systems, can be adopted to operate at biocompatible conditions [187], which make them good candidates for biofabrication of scaffolds with incorporated viable cells and/or bioactive molecules. Hydrogel-based scaffolds with designed patterns of encapsulated cells have been fabricated with AM techniques [89,90,91,188]. Designed patterns of growth factors immobilized to a biomaterial have also been precisely printed using an AM technique (*i.e*., inkjet printing) to study cellular response to engineered bio-guidance [189]. AM techniques have also been used to integrate nano-/micro-scaled features into scaffolds [69,190]. Microspheres or nanoparticles loaded with bioactive materials have been blended with backbone materials and dispenses by AM to make TE scaffolds [190]. These capabilities are of special interest for strategic cartilage TE because they can provide precise, design-based fabrication in biocompatible processing conditions. AM and indirect AM [191] techniques also have the potential to be integrated with other approaches for developing scaffolds with additional features and functionalities. For example, conventional porogen leaching and advanced AM techniques have been integrated to create scaffolds with both macro- and micro-features [69,192].

**Figure 4:**
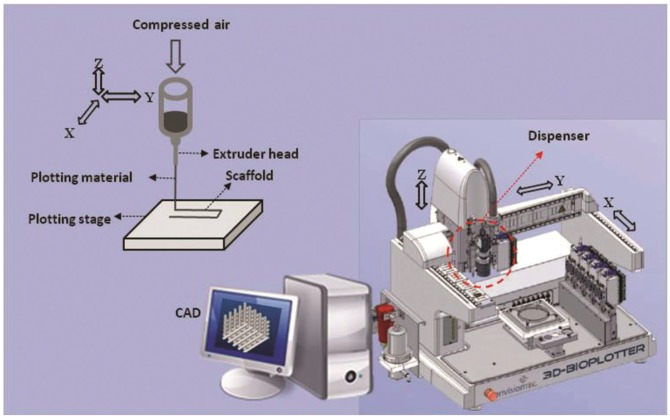
Schematic diagram of a 3D plotter additive manufacturing fabrication technique (Image courtesy of Envisiontec GmbH [210]).

3D plotting techniques (Figure 4) have become more common in TE [69,193,194] because a wide range of biomaterials (e.g., from polyester polymers to cell-/bioactive-laden hydrogels) can be employed during fabrication [195,196]. This is desirable for developing and studying complex scaffolds that have enhanced mechanical and biological functions for cartilage TE. Some of the important merits and demerits of 3D plotting for designed-based scaffold fabrication are listed in Table 3. Although theoretically possible, few studies have investigated the potential for building biomimetic designs and complexities into cartilage TE scaffolds using these fabrication techniques. In particular, the capability of 3D plotters for biofabrication of cartilage TE scaffolds (incorporating cells/bioactive molecules during fabrication) has received limited attention, yet could alleviate the low efficiency, nonuniform cell seeding issue that usually requires the additional step of dynamic seeding or culture [197,198,199]. Updated reviews of AM systems for TE of scaffolds, along with their detailed advantages and disadvantages, are available in the literature [187,200,201]. Some of the recent strategic designs of scaffolds investigated for cartilage TE using advanced fabrication techniques are reviewed in the following section.

**Table 3:** Merits and demerits of electrospinning and bioplotter fabrication techniques for design-based scaffold fabrication.

Merits and demerits	Electrospinning	Bioplotter-additive manufacturing
Merits	Fine fibers (25–100 µm), ECM-like structure (good for cellular activities) [34,106] Use of minimum amount of material [202], minimizing material–cell/tissue interaction [171] Potential biofabrication capacity [106,203] Capable of incorporating multiple polymers [204,205]	Reproducible fabrication [69,209] Computer controlled Building of designed, specified structures; patient-specific grafts [169,209] Processing the widest range of biomaterials: hydrogels to polymer melts and hard substances [85,196] Design-based biofabrication capacity [195]
Demerits	Densely packed structure, small pore size, nonuniform cell infiltration/tissue formation [206,207] Need of postfabrication process, e.g., direct perfusion [105] and dynamic culturing [173,208] Limited design-based architectural/properties	Limited at high spatial resolution [69,192]

## 4. Strategic Scaffold Designs for Cartilage Tissue Engineering

Recent efforts have been directed toward developing more functional, biomimetic scaffolds using designed structures, combined strategies, advanced fabrication techniques, and/or novel methodologies. This review focuses on the two strategies of hybrid and zonal TE scaffolds for cartilage repair.

### 4.1. Hybrid Scaffolds

Hydrogel and solid polymer backbone materials have advantages and disadvantages with respect to cartilage TE. However, the use of both materials in hybrid scaffolds can result in unique synergistic properties for better engineering of cartilage. In such hybrid designs, the polyester solid material provides a reinforcing skeleton for mechanical strength and the hydrogel provides a cell supportive/delivery matrix within the scaffold [1,28]. Composed of two essential components, hydrogel–solid hybrid scaffolds resemble the biphasic nature of articular cartilage (water and solid phases) and might be an effective strategy for TE cartilage repair. The superiority of hybrid scaffold over hydrogel- or solid-only scaffolds for cartilage TE has been demonstrated. Some reported advantages of hydrogels within solid scaffolds include retaining cells in the 3D, cell-friendly environment of the hydrogel, even distribution of cells in the solid scaffold pores, preventing cells from floating out of the scaffold, confining growth factors within the scaffold for better interaction with cells [211], and retaining the initial geometry (shape) of the scaffold. Synthetic PLGA mesh has been combined with chondrocyte-embedded fibrin glue to both preserve cell phenotype and provide controllable scaffold degradation properties [212]. The accumulation of GAGs after four weeks was 2.6 times more than in a pure PLGA scaffold [5]. Composite PLGA/cell-embedded alginate scaffolds enhanced expression of collagen type II [213]. An *in vivo* study of a cell-seeded collagen (type I)/PLGA scaffold showed homogeneous distribution of morphologically stable (round shape) cells and collagen type II formation [214]. Other similar examples in cartilage repair include combinations of PGA/alginate [215], PLA/alginate [216], PLGA/HA [217], and PLGA/fibrin [162,218]. 

**Figure 5:**
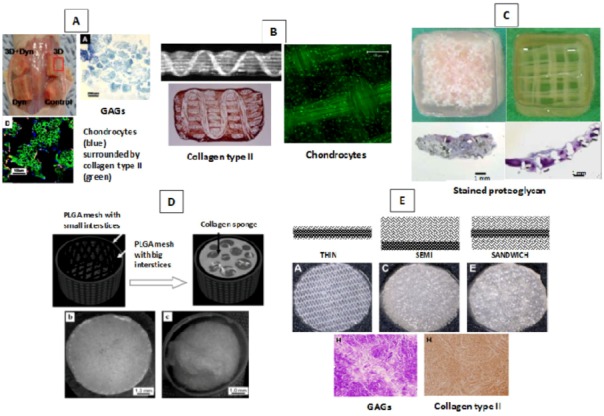
Different designs of hybrid scaffolds developed for cartilage TE: (**A**) PLCL-FG/HA [220,224]; (**B**) woven PGA/PCL-agarose/fibrin [46]; (**C**) PLLA-atelocollagen [110]; (**D**) PLGA-collagen [221]; and (**E**) PLGA-collagen [214].

Different approaches and designs have been investigated for creating hybrid scaffolds. Here, the most recent different designs for hybrid scaffolds in cartilage TE are reviewed. Infiltration of hydrogels into solid scaffolds has been very popular in cartilage TE. In brief, the method takes a solid scaffold framework and then loads it with hydrogel, which may or may not contain cells and/or bioactive molecules. The base solid framework is typically fiber/textile [87,112,212], sponge [162,211,216,218], or rapid prototyped structures [219]. The hydrogel loading procedure may be as simple as dropping the hydrogel cell suspension onto the solid scaffold and then molding the hybrid scaffold, or just seeding the solid scaffold with a hydrogel cell suspension [220]. Jung *et al*. [220] hybridized a synthetic poly (L-lactide-co-ε-caprolactone) (PLCL) polymer with chondrocyte-embedded fibrin gels (FG) and hyaluronan (HA) hydrogel. The porous solid framework was created by press-molding, salt leaching, and then freeze-drying of the PLCL-NaCl mixture. The chondrocyte-hydrogel suspensions were incubated in the PLCL scaffolds quickly after preparation. The solid PLCL framework (85% porosity and 300–500 µm pore size) possessed rubber-like elasticity, which could deliver stimulating mechanical signals to the cells. The round cellular morphology of the chondrocytes was properly retained in the inoculated hydrogels. Hybrid scaffolds made with higher viscosity hydrogels (*i.e.*, FG and HA) exhibited the highest cell adhesion efficiency among other created hybrid scaffolds. Overall, these observations indicate how the design of hydrogel properties can affect the scaffold biofunctionality. An eight-week *in vivo* study showed formation of a well-developed, homogeneously distributed cartilage construct that had comparable compression properties (0.57–0.77 MPa) to natural articular cartilage (Table 2) [220]; the fabrication method used did not allow any design-based control over the structural organization of the formed tissue, and ECM formed in the randomly distributed pores of the PLCL scaffold (Figure 5A).

Vacuum-assisted infusion of hydrogel/cell–hydrogel biomaterials is another approach that provides uniform infiltration of hydrogels into solid scaffolds (*i.e.*, dense structures) [46]. Moutos [46] hybridized an agarose/fibrin hydrogel with 3D woven PCL or PGA scaffolds (porosity of 70%~75%) using a vacuum-based infusion technique (Figure 5B). The design of a woven reinforcing component significantly improved the initial mechanical properties (tensile and compression) of the hybrid scaffold to within the range of natural articular cartilage. The structural design of the hybrid scaffold provided biomimetic mechanical properties including anisotropy, viscoelasticity, and tension-compression nonlinearity. The hydrogel component was observed to improve viscoelastic creep behavior and stiffness. However, the highly intense design of the solid framework resulted in the accumulation of ECM around the perimeter of the hybrid scaffold (Figure 5B) and lower mechanical properties in the engineered construct [46]. 

Tanaka *et al*. [110] systematically studied the effect of design (architecture and composition) on the functionality of atelocollagen/synthetic polymer hybrid scaffolds. Two groups of sponge and nonwoven fibrous poly-L-lactide acid (PLLA) scaffolds were fabricated, with each design created at different pore sizes and porosities (Figure 5C). Chondrocyte-embedded atelocollagen was injected into the porous solid scaffolds, incubated for gelation, and then the scaffolds subcutaneously implanted *in vivo*. Retention of the cell–atelocollagen mixture was highest in the scaffolds with the highest pore sizes and porosities. Sponge scaffolds show higher retention of the hydrogel than fibrous scaffolds, mainly due to their generally higher porosity, at 90%–95% compared to 85%–90%. The hybrid scaffold designs that demonstrated superior accumulation of collagen type II and GAGs include sponge-based scaffolds, with a pore size of 0.3 mm and porosity of 95%, and fibrous-based scaffolds, with a pore size of 1.5 mm and porosity of 88%. Collagen type I was generally higher in fibrous-reinforced *vs.* sponge-reinforced constructs. Accumulation of macrophages was also observed on and around the polymeric part of the hybrid scaffolds [110]. Unfortunately, the mechanical properties of the hybrid scaffolds and TE constructs were not investigated despite this information being of great value for cartilage TE applications. One limitation of fibrous-reinforced scaffolds is the large fiber size (smallest achievable diameter of 1 mm), which inhibits fabrication of scaffolds with higher porosities and consequently inhibits formation of larger tissue volumes. Advanced AM fabrication techniques can address this issue through higher achievable resolutions [110].

Kawazoe *et al*. [221] developed a PLGA-collagen hybrid scaffold for cartilage repair that was specifically designed to prevent the cells from leaking out of the scaffold during seeding. The design included a bilayered, cup-shaped mesh membrane of PLGA filled with collagen sponge (freeze-dried collagen) (Figure 5D). Knitted and woven PLGA meshes, with big and small interstices, respectively, were glued to each other to maintain the 3D structure of the collagen (to prevent shrinking) and protect against cell leakage. The developed hybrid scaffold had comparable mechanical properties (e.g., compression Young’s modulus) to articular cartilage and was successful in preventing cell leakage, having a cell seeding efficiency four times higher than a nonhybrid scaffold. This and similar hybrid scaffold designs [114,222,223] still require postfabrication cell seeding, which may involve noncontrollable/-reproducible cell infiltration and distribution. 

Dai *et al*. [214] developed three designs of hybrid PLGA-collagen scaffolds for cartilage TE: a knitted PLGA mesh with collagen sponge in its interstices (called THIN), a knitted PLGA mesh with 3 mm collagen sponge on one side (called SEMI), and a knitted PLGA mesh with 3mm collagen sponge on both sides (called SANDWICH) (Figure 5E). Scaffolds were seeded with chondrocytes after fabrication and transplanted *in vivo*. Cell seeding efficiencies were higher in SEMI and SANDWICH designs than the THIN design. Two to eight weeks after transplantation, cartilage-like tissues that formed in SEMI and SANDWICH designs were thicker and higher in GAGs and collagen type II than in the THIN design. The maximum achieved Young’s modulus and stiffness was associated with the SEMI design; 54.8% and 68.8%, respectively, of that of natural cartilage [214]. This indicates how the initial design of the hybrid scaffold can affect the mechanical properties of the engineered construct. No significant difference was reported between the mechanical and biochemical properties of the constructs formed by SEMI and SANDWICH designs. 

Comparison of the appearance of the tissue formed in different hybrid scaffold designs (Figure 5) shows the influence of scaffold design on the structural organization of formed tissue. Despite the number of hybrid scaffolds tested for cartilage TE, few studies have investigated the development of hybrid scaffolds with reproducible solid and hydrogel components throughout the scaffold. More specifically, the customized spatial distribution of hydrogels and/or cells and varying the composition of hydrogels throughout hybrid scaffolds, have not yet been investigated for cartilage TE. 

**Figure 6:**
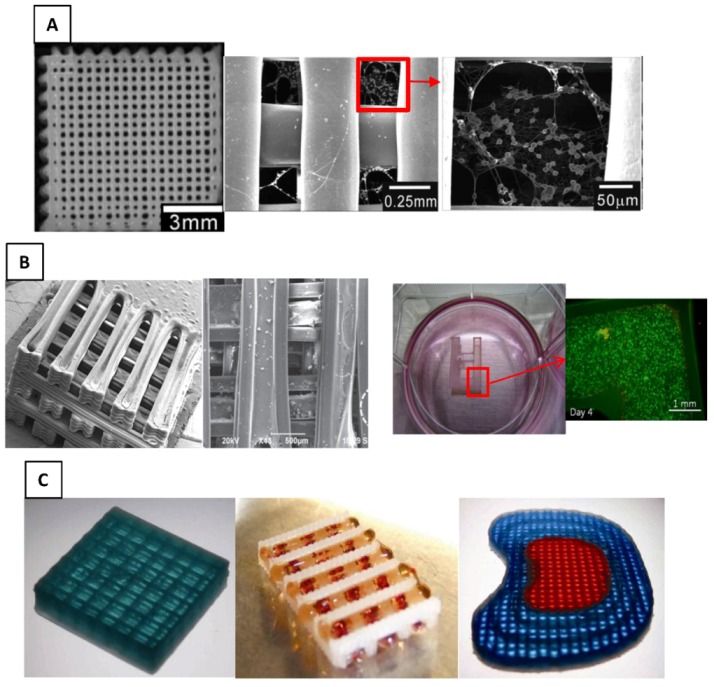
Controlled deposition hybrid scaffolds: (**A**) PCL-electrospun collagen [225]; (**B**) PCL/PLGA-hydrogel [196]; and (**C**) PCL-alginate [195].

Recently, hybrid scaffolds with controlled spatial distribution of hydrogels and solid polymers have been developed using advances in fabrication techniques. Although these studies were not specifically aimed at cartilage TE, they shed light into potential advances that could be achieved in cartilage TE. Lee *et al*. [225] combined AM and electrospinning fabrication techniques to develop hybrid scaffolds from PCL and collagen biomaterials. Melted PCL was plotted in two layers of perpendicular strands and then collagen nanofibers electrospun on top of the PCL strands. This pattern was repeated to make a 3D hybrid scaffold (Figure 6A), which was later seeded with cells. This hybrid scaffold showed better mechanical properties (tensile Young’s modulus) and biological activity than a pure PCL scaffold. Although not tested by Lee *et al*. [225], electrospinning could be used to incorporate viable cells during fabrication [203] and, therefore, achieve the design of a biological hybrid scaffold. 

Shim *et al*. [196] employed a multihead deposition system to create a hybrid scaffold from synthetic polymers and hydrogel. A mixture of PCL-PLGA was deposited in three layers (nonperpendicular pattern), then hydrogel (hyaluronic acid, gelatin, and atelocollagen) infused into the canals created between the solid strands. The same pattern was repeated to produce a 3D hybrid scaffold (Figure 6B). Using this fabrication technique, the dispensing position of both synthetic polymer and hydrogel inside the scaffold was exactly controlled. Shim *et al*. [196] also used this fabrication process to make one layer of a hybrid scaffold with a cell-laden hydrogel. The viability of the dispensed cells was 97.8% and 94.8% at 4 and 10 days after dispensing, respectively, which indicates that cell printing may not affect cell viability. Due to limitations of the fabrication process, only one cell-embedded layer was included in the hybrid scaffold (Figure 6B). Practical challenges of the fabrication process that may influence the biological integrity of the hybrid scaffold were not addressed, including use of toxic organic solvents during fabrication. 

Schuurman *et al*. [195] created 3D hybrid scaffolds from CAD models using PCL and a cell-embedded alginate hydrogel, demonstrating the feasibility of making hybrid scaffolds with customized shapes, internal architectural designs, and depth-varying hydrogel materials (Figure 6C). Viability of cells in this type of hybrid scaffold, three days after fabrication, was reported to be within the same range as those in a nonprinted, hydrogel-only scaffold. The Young’s modulus of the developed hybrid scaffold was within the range of natural articular cartilage. The results of these studies confirm the feasibility of using advanced fabrication techniques to develop multiphase hybrid scaffolds with controlled and designed distribution of cells, hydrogel, and synthetic materials. These strategies and fabrication techniques could be employed for developing more functional cartilage TE scaffolds that have customized biological and mechanical properties. 

### 4.2. Zonal Scaffolds

The exceptional mechanical functionality of natural articular cartilage can be attributed to the distinctive organization of cartilage into four zones from the articular surface down to subchondral bone. As discussed in the introduction, the variation in cell morphology and ECM matrix composition/structure across these regions is believed to be a key factor in the mechanical functionality of the cartilage. One problem with current engineered cartilage constructs is the absence of normal cell and ECM zonal organization, which is critical to natural cartilage function. Recently, researchers have attempted to replicate this zonal variation in scaffold design to improve the functionality of the resulting TE cartilage constructs. Cartilage TE scaffolds with a zonal design, as described by Klein *et al*. [21], can be categorized into either scaffold-free (cell-based) or scaffold-based approaches. The scaffold-based approach may use zonal solid, hydrogel, or hybrid scaffolds. Some efforts have been made to design scaffolds that promote engineered cartilage with zonally specific structure/function. 

In a scaffold-free approach, zonal chondrocytes, which have been isolated from different zones of natural cartilage, are zonally layered to replicate the normal zonal organization of cells. This approach can induce cartilage ECM with a structure on the upper surface similar to the superficial zone of natural cartilage [115,226,227]. Self-assembling fabrication techniques [228] could potentially facilitate scaffold-free approaches for zonal cartilage TE. Although the initial mechanical properties of such cell-based structures are low, strategies can be taken to improve the mechanical properties of the self-assembled TE constructs [228,229,230,231]. Advances in AM technology (*i.e.*, bioprinting self-assembly) [232] could significantly contribute to the design and customization of physical/biochemical properties of assembled structures. One limitation of zonal cell-based TE is the difficulty in isolating cells from separate zones in articular cartilage, especially in human cartilage [61].

Zonally varying structural/biochemical designs in solid scaffolds is another approach for replicating cartilage zonal organization. Oriented nanofibrous scaffolds can induce cartilage tissue with oriented mechanical and cellular properties similar to the superficial zone of natural cartilage [233]. Cartilage TE scaffolds with designed zonally varied physical parameters (no variation in biological properties) [61] can mimic the anisotropic distribution of cells but not the zonal ECM organization. Natural and synthetic hydrogels with zonal-varying properties have also been investigated for cartilage zonal TE [234,235,236,237,238,239]. Zonal chondrocytes have been successfully expanded/cultured separately in hydrogels for zonal cartilage TE [238,240,241]. Zonal hydrogel scaffolds with a spatial gradient of embedded chondrocyte concentration [43] or type (zonal chondrocytes) [242,243] have been developed using a multilayer scaffold design. The interaction between superficial and deep/middle zone chondrocytes in a bilayer scaffold design resulted in depth-varying cellular activity and heterogeneous ECM formation. A depth-dependent compression modulus [242] and enhanced mechanical properties (shear and compression strength) [243] have been observed in zonal constructs when compared to single-layer hydrogel scaffolds. Nguyen *et al*. [239] designed a hydrogel with zonally varying biochemical composition that can drive mesenchymal stem cells into zone-specific chondrocytes and promote zone-specific chondrogenesis similar to natural cartilage. The developed hydrogels [239] are intended to be incorporated into a layered hydrogel scaffold for zonal cartilage TE. In this type of zonal scaffold, the hydrogel for each layer is prepared separately and then assembled to make a multilayer, zonal scaffold. One limitation of this approach is the delamination of distinct layers by shear stress of applied loads. Efficient layering strategies, which ensure sufficient integration/stability of these heterogeneous biomimetic gels, are required to address this issue. 

In a very recent study, Fedorovich *et al*. [88] investigated the use of AM fabrication technique for making cell-laden heterogeneous (layered) hydrogel scaffolds for cartilage TE. Two different hydrogel layers with different cell types were successfully fabricated into a 3D zonal scaffold. Hydrogel scaffolds designed with varying architectural (*i.e.*, fiber spacing, angle, and porosity) and mechanical properties (*i.e.*, elastic modulus) have been successfully fabricated and tested both *in vitro* and *in vivo* [88]. A method has also been developed for engineering multiphase structures with controlled and designed properties in each phase, as well as at the interfaces [244]. More specifically, multiple phases of collagen fibers, the density and size of which were modulated by varying collagen concentration and gelling temperature, were integrated into an engineered structure. Using this technique, significant adhesion strength was achieved at the interfaces of the multiphase construct [244], which could be important for layered cartilage TE design. 

Hybrid scaffolds have also been designed and created to promote the formation of a zonal cartilage–bone interface (continuous gradations in formed ECM) [88,245,246]. A review of current strategies for developing TE osteochondral (bone–cartilage) grafts is given by O’Shea and Miao [247]. The effectiveness of bioreactors and mechanical loadings for stimulating the deposition of zone-specific cartilage ECM in TE scaffolds has also been demonstrated [248,249,250]. A thorough review of zonal cartilage TE strategies can be found in Klein *et al*. [21,251].

## 5. Conclusions and Recommendations for Future Research

During the last 20 years, significant advances have been made in cartilage TE with respect to scaffold biomaterials, design, and fabrication. Hopes have been increased with respect to the development of a tissue engineering-based treatment for osteoarthritis, as well as cartilage lesions in young patients. This review discussed design considerations along with techniques used for fabrication of designed scaffolds for cartilage TE. Progress and advances in two strategic designs of hybrid and biomimetic zonal scaffolds were also reviewed. Although encouraging progress has been made, the major challenge in TE cartilage repair remains the insufficient resemblance of engineered cartilage to natural hyaline cartilage, in terms of biochemical composition, structural organization, and biomechanical properties [252]. To systematically improve this limitation using bioengineered scaffolds, strategic scaffold designs should be developed and investigated toward creating more functional cartilage TE constructs. One approach could be to learn from previous designs, with the mechanisms and/or factors that are currently inhibiting successful formation of functional cartilage tissue investigated and addressed in scaffold design and/or therapeutic strategies. Another approach is to develop and investigate biomimetic scaffold designs using knowledge from natural cartilage tissue systems and/or the healing process. This could include introduction of biological (e.g., bioactive molecules/cells) and structural (e.g., ECM-like architecture) complexities, similar to natural cartilage, into the scaffold design. Using the available advanced fabrication techniques, highly complex and designed scaffolds with a biomimetic distribution of cells and/or bioactive molecules within the scaffold could be created and investigated for design-based functional cartilage TE. Furthermore, different designs that have been successfully developed could be integrated and combined into new scaffold designs to achieve improved functionality for better cartilage TE. A current example of this approach is hybrid scaffolds with a hydrogel formulation designed for successful chondrogenesis and a solid framework designed for sufficient biomechanical functionality and formed tissue affinity. Hybrid scaffolds with biomimetic (e.g., zonal) designs are another interesting option to be investigated for cartilage TE; the solid framework structure and cell–hydrogel formulation could be designed to vary at different zones of a hybrid scaffold to achieve biomimetic zonal properties and signals. Biomimetic zonal cartilage TE is still in its infancy and more work in the development of design and fabrication methods is required to obtain zonally tailored TE scaffolds that promote zonal cartilage tissue formation. Furthermore, whether or not a biomimetic zonal design will actually result in functional (mechanically and/or biologically) engineered cartilage, as compared to a nonzonal TE strategy, remains to be determined [21].

Although available advanced fabrication techniques have several benefits and capabilities, limited work has been done to investigate scaffolds designed with built-in biological/structural properties for cartilage TE; this should be conducted in future cartilage TE studies. More complex scaffold designs may not be easily achieved and studied without sophisticated fabrication techniques. Advancements in fabrication techniques for facilitating the development of precisely designed bioengineered scaffolds are urgently needed. Specifically, programmable, multifunctional, computer-controlled biofabrication techniques with higher resolution (*i.e.*, smaller fiber diameter and spacing) could significantly contribute to advances in cartilage TE. The reproducibility of scaffolds in a sterile, nontoxic environment could reduce the steps required for scaffold preparation and thus accelerate the economical transition of cartilage TE strategies to clinical applications. Fabrication systems that are able to facilitate this transition, by accommodating multiple functions, are of great interest with respect to development and commercialization. Some efforts have been made to develop integrated fabrication systems that prepare cell-incorporated biological samples [253] or TE scaffolds [254] in a sterile environment, including the nanoplotter-laminar flow hood [253] and stereolithography system-laminar flow hood [254]. Ongoing advances in technology and instrumentation could significantly contribute to this task.

Although the effect of different scaffold properties on chondrogenesis has been widely investigated, the exact mechanisms by which these properties affect neocartilage structure/composition are not fully understood. Investigating the rationale behind this cause and effect relationship will help to efficiently optimize scaffold design parameters and achieve better results. Developing mathematical models that can simulate the tissue formation process and subsequently relate scaffold properties to engineered tissue properties would be of special interest and benefit to scaffold optimization. One shortcoming in the cartilage TE literature is the lack of consistent mechanical and biological tests and/or evaluation criteria, which inhibits valid comparisons of designs and strategies. Most of the results reported are based on a variety of different testing conditions and criteria, and thus the development of standard evaluation protocols for mechanical and biological assessment of cartilage TE constructs will be beneficial for the comparison of different studies and the development of better strategies. 

Different results observed from *in vivo* and *in vitro* studies of the same scaffold design demonstrate the need and importance of *in vivo* studies for better understanding real-life scaffold performance. Published *in vivo* studies are considerably fewer in number than *in vitro* studies; this issue becomes especially important when meticulously designed scaffolds, based on *in vitro* tests, perform differently *in vivo* [255,256]. Because the *in vivo* environment is where the scaffold will finally perform [21], cartilage TE studies should move toward more *in vivo* experiments. Sterilization techniques and their influence on the integrity of TE scaffolds (e.g., scaffold material mechanical properties) should also be given more attention in scaffold design. Secure fixation of the engineered construct or TE scaffold in the transplantation site, which will be highly load-bearing joint, is another issue that should be considered in the design and/or fabrication of TE scaffolds/constructs. Weak or inappropriate implantation may significantly affect neocartilage formation, structural organization, integration with host tissue, and its remodeling. 
